# Angiotensin II type 2 receptor activation preserves megalin in the kidney and prevents proteinuria in high salt diet fed rats

**DOI:** 10.1038/s41598-023-31454-6

**Published:** 2023-03-15

**Authors:** Kalyani Kulkarni, Sanket Patel, Riyasat Ali, Tahir Hussain

**Affiliations:** Department of Pharmacological and Pharmaceutical Sciences, College of Pharmacy, University of Houston, Health 2, 4349 Martin Luther King Boulevard, Houston, TX 77204-5037 USA

**Keywords:** Diseases, Nephrology

## Abstract

Proteinuria is a risk factor for and consequence of kidney injury. Angiotensin II type 2 receptor (AT_2_R) is an emerging reno-protective target and is anti-proteinuric under pathological conditions, including high salt-fed obese animals. However, the mechanisms remain unknown, particularly whether the anti-proteinuric activity of AT_2_R is independent of its anti-hypertensive and anti-inflammatory effects. In the present study, obese Zucker rats were fed high sodium (4%) diet (HSD) for 48 h, a time in which blood pressure does not change. HSD caused proteinuria without affecting glomerular slit diaphragm proteins (nephrin and podocin), glomerular filtration rate, inflammatory and fibrotic markers (TNFα, IL-6, and TGF-β), ruling out glomerular injury, inflammation and fibrosis but indicating tubular mechanisms of proteinuria. At cellular and molecular levels, we observed a glycogen synthase kinase (GSK)-3β-mediated megalin phosphorylation, and its subsequent endocytosis and lysosomal degradation in HSD-fed rat kidneys. Megalin is a major proximal tubular endocytic protein transporter. The AT_2_R agonist C21 (0.3 mg/kg/day, i.p.) administration prevented proteinuria and rescued megalin surface expression potentially by activating Akt-mediated phosphorylation and inactivation of GSK-3β in HSD-fed rat kidneys. Overall, AT_2_R has a direct anti-proteinuric activity, potentially via megalin regulation, and is suggested as a novel target to limit kidney injury.

## Introduction

Proteinuria/albuminuria is a consequence and a risk factor for chronic kidney disease (CKD) under various pathological conditions, mainly hypertension and diabetes. Recently, angiotensin II type 2 receptor (AT_2_R), a component of the protective arm of the renin angiotensin system, has been reported as reno-protective, anti-proteinuric, anti-inflammatory, anti-fibrotic and anti-hypertensive, including in high salt diet (HSD)-fed animals^1–11^. Generally, these studies were chronic and the anti-proteinuric effects of AT_2_R were associated with a reduction in blood pressure. Specifically, our laboratory has reported that obese Zucker rats (OZR) when fed with HSD for 14 days exhibited proteinuria and tubulointerstitial injury, which were attenuated by AT_2_R activation^1^. Since high salt-intake induces inflammation, fibrosis and causes an increase in blood pressure, particularly in obesity^1,12^, these processes could be responsible for proteinuria and kidney injury. Considering the opposing effects of AT_2_R and high salt-intake, it is unknown whether anti-proteinuric effects were due to anti-hypertensive and anti-inflammatory effects of AT_2_R or a direct action related to AT_2_R activation in HSD fed animals. Therefore, present study is designed to elucidate the anti-proteinuric novel mechanism of AT_2_R activation independent of changes in blood pressure, inflammation, and fibrosis.

Tubular proteinuria is characterized as an impaired or reduced uptake of glomerular filtrate proteins by proximal tubular epithelial cells^13–16^. Megalin mediated endocytosis is an important function of proximal tubule epithelial cells to reabsorb proteins filtered through glomerulus. Megalin, a low-density lipoprotein receptor, is expressed abundantly on the apical surface of the proximal tubule epithelial cells and is a fast-recycling endocytic receptor. There is evidence that megalin is phosphorylated by GSK3β ^17,18^ and GSK3β -induced phosphorylation negatively regulates receptor recycling and reduces cell surface expression of the receptor^18^. Megalin is endocytosed after binding to filtered proteins and is recycled back to the plasma membrane after directing the bound proteins to lysosomal degradation^17,19–23^. Cubilin is another endocytic protein expressed on the plasma membrane^24–26^. Under normal physiological conditions, these receptors are responsible for tubular clearance of low and high molecular weight proteins and are recycled back to the plasma membrane^25,26^. Reduction in the functional activity of megalin/cubilin leads to proteinuria, which, if not resolved, can lead to inflammation, fibrosis, kidney injury culminating into cardiovascular diseases, in the long-term^20,21^. The AT_2_R is linked to Akt phosphorylation (activation)^27^ and GSK3β is inactivated upon phosphorylation by Akt^28^. Considering that GSK3β induces phosphorylation of megalin and negatively regulates its recycling reducing cell surface expression^29,30^, we hypothesize that AT_2_R-mediated activation of Akt reduces GSK3β activity and megalin phosphorylation, which in turn protects cell surface megalin expression and reduces proteinuria induced by high salt-intake. This hypothesis was tested in obese rats fed high sodium diet and administered with the AT_2_R novel agonist compound-21 (C21), which is a novel agonist and well-studied for its specificity and efficacy for various AT_2_R-mediated function, including human clinical studies^27^.

## Methods

The Institutional Animal Care and Use Committee at University of Houston approved these protocols.

### Ethical approval

All methods in this study are reported in accordance with the ARRIVE guidelines (https://arriveguidelines.org/) that maximize the quality and reliability of published research, and enabling others to better scrutinize, evaluate and reproduce it. Humans are not involved in this study. All the methods were carried out in accordance with relevant guidelines and regulations.

### Animals

Male OZR, 11–14 weeks old were purchased from Envigo, Indianapolis. The animals were acclimatized for a week at the University of Houston animal care facility upon arrival. In vivo experimental protocols used in this study were approved by the Institutional Animal Care and Use Committee at the University of Houston. The animals were fed with NSD (0.4%; TekLad TD.99215, Harlan laboratories) or HSD (4%, TekLad TD.92034, Harlan laboratories) and treated with AT_2_R agonist C21 (0.3 mg/kg/day i.p.) for 2 days. The specificity of C21 in vivo as well as in vitro studies in our laboratory has been tested by blocking its effects with the AT_2_R antagonist PD123319^4,7^. Rats were placed in metabolic cages during the study for urine collection. Body weight, food intake, water consumption and urine output were measured at 24 and 48 h. At the end of the study, blood was collected through cardiac puncture under isoflurane (2–3%) anesthesia, processed for plasma, and stored at − 80 °C. Kidney cortices were collected and a part of it was embedded in OCT and stored at − 80 °C.

### Proteinuria and albuminuria

Urinary protein was measured by pyrogallol red (PR)-molybdate method. Briefly, to 5 μL of centrifuged urine sample, 200 μL PR-molybdate reagent was added and allowed to react for 10 min at 37 °C. Absorbance was read at 600 nm to measure total protein (mg/mL). Urinary albumin was determined by Nephrat II competitive ELISA kit (catalog# NR002 Ethos biosciences) according to manufacturer’s protocol. Urinary protein and albumin were normalized with urine volume (mL/hr) and reported as excretion rate in mg/hr.

### Immunoblotting

The expression of podocin, nephrin, pAkt and pGSK3β in the kidney cortices was determined by western blot analysis. Equal amount of protein (20 μg for podocin and nephrin, and 100 μg for pAkt and pGSK3β) was loaded at 4–20% SDS-PAGE, transferred to activated PVDF membrane, and immunoblotted with anti-podocin, anti-nephrin, anti-phospho-Akt (Ser-473) and anti-phospho-GSK3β (Ser9) respectively. β-Actin was used as loading control for podocin and nephrin and total Akt and total GSK3β were used to normalize  pAkt and pGSK3β. For dot blot analysis, an equal amount of protein (10 μg) was directly spotted onto the activated PVDF membrane. The membrane was then incubated with specific anti-megalin or anti-cubilin antibody in 5% BSA-PBST (phosphate buffered saline containing 0.05% tween-20) overnight at 4 °C. The membrane was washed with PBST (5 mL, 10 min × 3), immunoprobed with relevant secondary HRP-conjugated antibodies, namely goat-anti-mouse IgG secondary antibody, goat-anti-rabbit IgG secondary antibody, for 1 h at room temperature, washed with PBST and the electrochemiluminescence signal was recorded and the bands density was analyzed (BioRad ChemiDoc MP Imaging System or Li-Cor Odyssey Fc Imager). The original blots are provided in SI Fig. S1-4.

### Separation of phospho-megalin, phospho-Akt and phospho-GSK3β

To determine the phosphorylated proteins, SuperSep Phos-tag (50 μmol/l), 7.5%, 17 well, 83 × 100 × 3.9 mm (FUJIFILM Wako Pure Chemical Corporation catalog# 198–17,981) was used. Phos-tag gel is a novel method which separates phospho-proteins based on migration and band shift which relies on complex formation ability. Phospho-proteins separate at a higher level compared with the non-phosphorylated form. Phos-tag allows to study phospho-proteins independent of phospho-specific antibody. Moreover, the stripping procedure is not required, hence, this is the method of choice to study phospho-proteins independent of loading control (e.g., GAPDH, beta-actin, etc.). The samples were prepared using RIPA lysis buffer without EDTA (150 mM NaCl, 1% NP-40, 1% sodium deoxycholate, Tris–HCl and 1% SDS with protein phosphatase inhibitor) and samples were separated for 9 h. at 10 mA. Before transferring the gel on the PVDF membrane, the gel was washed with transfer buffer containing 10 mmol/L EDTA and 3 times for 10 min each. The gel was then immersed in transfer buffer without EDTA for 10 min. Transfer buffer without EDTA was used to transfer the proteins on the PVDF membrane. Immunoblotting with megalin antibody, Akt or GSK3β antibody was performed traditionally as explained earlier. The chemiluminescence signal was recorded and analyzed densitometrically by BioRad ChemiDoc MP Imaging System or Li-Cor Odyssey Fc Imager. The data is represented as the density ratio of the phospho- to non-phospho bands. The original blots are provided in SI Fig. S5. The validation of antibody by comparing the binding of boiled (vs. unboiled) antibody along with the specificity of secondary antibody is provided (SI Fig. S6).

### Immunofluorescence and colocalization

Approximately 20 μm thick sections were used for immunofluorescence experiment. The sections were incubated and permeabilized/blocked with 0.4% BSA, 0.2% saponin and 1% of the animal serum (donkey) in which the secondary antibody is raised in 1X PBS for 1 h. at room temperature. This blocking buffer was discarded and 1X PBS containing the anti-megalin, anti-cubilin antibody and/or anti-LAMP1 in 0.2% BSA and 0.1% saponin was added to the sections and incubated overnight at 4 °C. The sections were washed with 1X PBS (10 min × 4) and secondary antibodies for megalin, cubilin, and/or LAMP1 in 1X PBS containing 0.2% BSA and 0.1% saponin was added and incubated for 2 h. at room temperature. The sections were washed (10 min × 4) with 1X PBS and incubated with DAPI (catalog# D1306, Thermo Fisher Scientific, 1:3000, 5 mg/mL stock) for 10 min followed by washing 3-times with PBS. The sections and coverslip were mounted on slides with glycerol and were imaged using Leica confocal microscope (DMi8). We have acquired images at the depth of 1 micron Z stacks (pinhole 1 AU, 63 × objective lens, HyD or PMT detector, pixel format/dimension 1024 × 1024; sequential scanning mode). Moreover, we have confirmed the membrane vs cytosolic fluorescence signal of megalin by XZ or YZ image planes (Fig. S7).

### Creatinine and GFR measurements

Urinary creatinine was measured using BioAssay systems kit (catalog# DICT500) according to the manufacturer’s protocol. The data was reported as mg/day. Plasma creatinine was measured by Arbor Assays kit (catalog# KB02-H1) according to the manufacturer’s protocol. The values were reported as mg/dL. The GFR was calculated creatinine clearance method.

### Atomic absorption spectroscopy

This method was used to measure urinary and plasma sodium. The standards and samples were prepared according to the company’s protocol and the data was calculated using Beer’s law.

### Quantitative RT-PCR analysis for mRNA expression

Total RNA from frozen kidneys was extracted using the RNAEasy kit (Qiagen) according to the manufacturer’s protocol. A total of 500 ng of RNA were reversed transcribed into cDNA using ReverTra Ace qPCR RT Master Mix with gDNA remover (Diagnocine). This cDNA was used to semi-quantitate cytokines (TNF-α, IL-10, TGF-β, megalin and cubilin) using Thunderbird SYBR qPCR master mix (Diagnocine) in CFX Connect RT-PCR (Bio-Rad). Specific quantitative PCR primers for TNF-α (catalog# RP300044), TGF-β (catalog# RP300111), and IL-6 (catalog# RP300072) were purchased from Sino Biological, and megalin (catalog# 316,614,765 F [Sequence: TGG AAT CTC CCT TGA TCC TG], catalog# 316,614,766 R [Sequence: TGT TGC TGC CAT CAG TCT TC]) and cubilin (catalog# 316,614,763 F [Sequence: GCA CTG GCA ATG AAC TAG CA], catalog# 316,614,764 R [Sequence: TGA TCC AGG AGC ACT CTG TG]) from Integrated DNA Technologies. Expression of each gene was normalized to β -actin (catalog# VRPS-97, Real Time Primers, LLC), and relative fold expression values were calculated using a DD threshold cycle method.

### Chemicals

Anti-podocin (Santa Cruz, catalog# sc-518088), anti-nephrin (Santa Cruz, catalog# sc-377246), anti-phospho-Akt (Ser-473) (Cell Signaling, catalog# 9271), anti-phospho-GSK3β (Ser9) (Cell Signaling, catalog# 9336), β-actin (Santa Cruz, catalog# sc-47778), total Akt (Cell Signaling, catalog# 9272), total GSK3β (Cell Signaling, catalog# 9315), anti-megalin (Santa Cruz, catalog# sc-515750), anti-cubilin antibody (Santa Cruz, catalog# sc-518059), anti-LAMP1 (Development Studies Hybridoma Bank, catalog# 1D4B), Alexa fluor 488 anti-mouse for megalin, Alexa fluor 488 anti-rabbit for cubilin, Alexa fluor 568 anti-rat for LAMP1.

### Statistical analysis

The data were analyzed using GraphPad Prism Version 9.1.2 (225). Data are represented as mean ± sem. Statistical analysis was performed using one-way or two-way ANOVA with Fisher’s LSD for multiple comparisons and *p < 0.05 versus NSD and #*p* < 0.05 versus HSD considered statistically significant.

## Results

### Body weight

The body weight of animals among the study groups remain unchanged (NSD: 576 ± 23 g; HSD: 586 ± 22 g; HSD + C21: 596 ± 17 g).

### Renal function parameters

**Figure 1:**
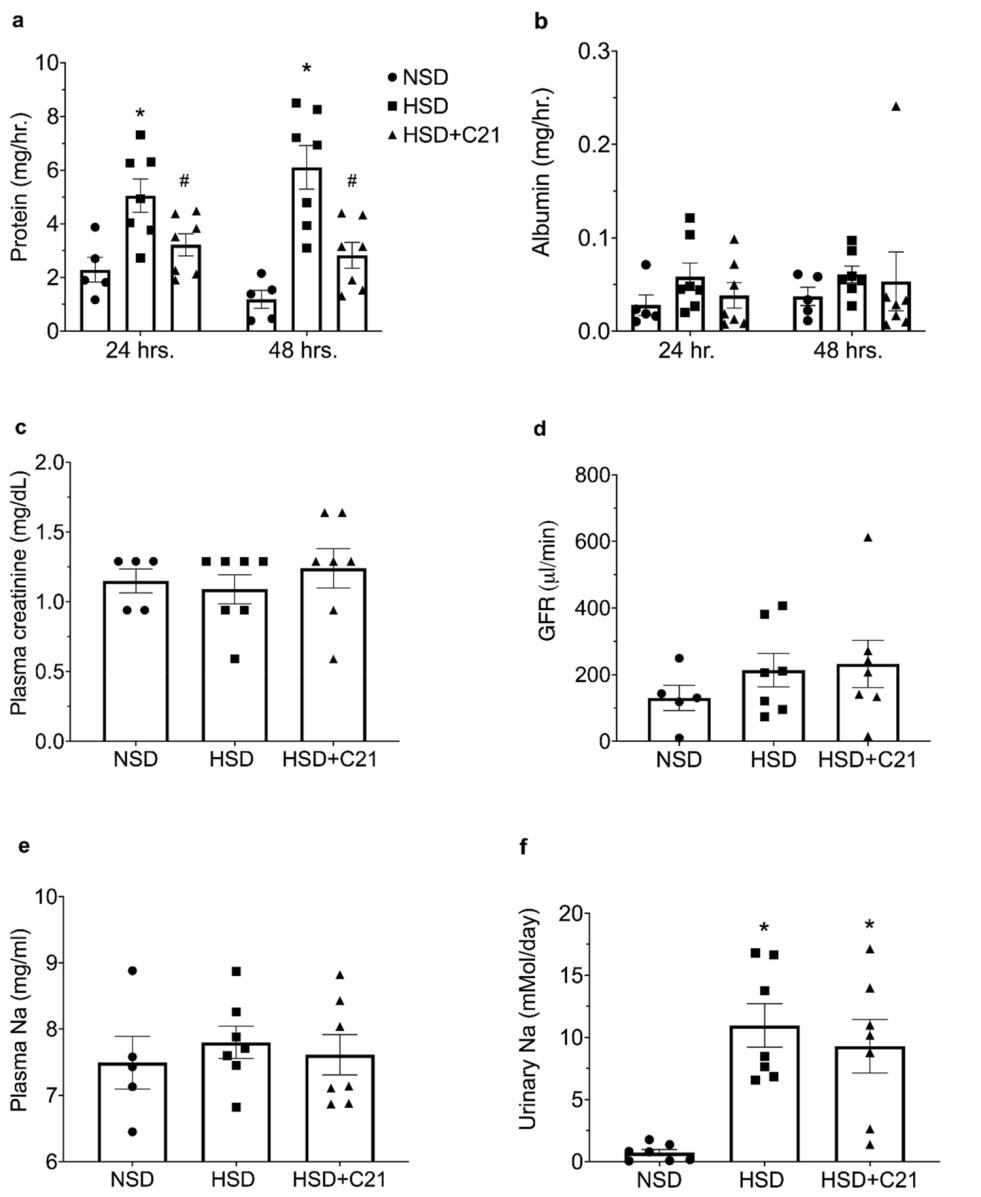
Effect of C21 treatment on renal function parameters of OZR fed with HSD or HSD + C21 for 48 h. Total urinary protein (proteinuria; **a**), total urinary albumin (albuminuria; **b**), plasma creatinine (**c**) and GFR (**d**). HSD and HSD + C21 treatment had no effect on plasma sodium concentration (**e**) however, HSD and HSD + C21 treatment groups exhibited a significant amount of sodium excretion in the urine as compared to NSD group (**f**). The values are represented as mean  +  sem; two-way ANOVA and one-way ANOVA followed by Fisher’s LSD test. *p < 0.05 versus NSD and #*p* < 0.05 versus HSD.

Urinary protein excretion was found to be increased by HSD feeding for 24 h. (HSD: 5.0 ± 0.6 mg/hr. vs. NSD: 2.2 ± 0.4 mg/hr.) and 48 h. (HSD: 6.1 ± 0.8 mg/hr. vs. NSD: 1.1 ± 0.8 mg/hr.). C21 treatment significantly reduced urinary protein excretion for both 24 and 48 h. respectively as compared to HSD feeding (HSD + C21: 3.2 ± 0.4 mg/hr. vs. HSD: 5 ± 0.6 mg/hr. at 24 h. and HSD + C21: 2.8 ± 0.4 mg/hr. vs. HSD: 6.1 ± 0.8 mg/hr. at 48 h.; Fig. 1a). However, urinary albumin excretion remained unchanged in HSD fed rats when compared to NSD group and C21 treatment also did not show any effect at both the time points (Fig. 1b). HSD feeding and C21 treatment did not cause any change in the plasma creatinine (Fig. 1c) and GFR in obese rats. (Fig. 1d).

### Plasma and urinary Na^+^ concentration

Compared with that in the NSD group, the plasma Na^+^ concentration in the HSD and HSD + C21 group did not change, as expected (Fig. 1e) but HSD and HSD + C21 fed rats excreted significantly higher amount of urinary Na^+^ (Fig. 1f; HSD: 10.9 ± 1.7 mMol/day vs. NSD: 0.7 ± 0.2 mMol/day; HSD + C21: 9.29 ± 2.1 mMol/day vs. NSD: 0.7 ± 0.2 mMol/day), compared with NSD rats.

### Expression of endocytic receptors

**Figure 2:**
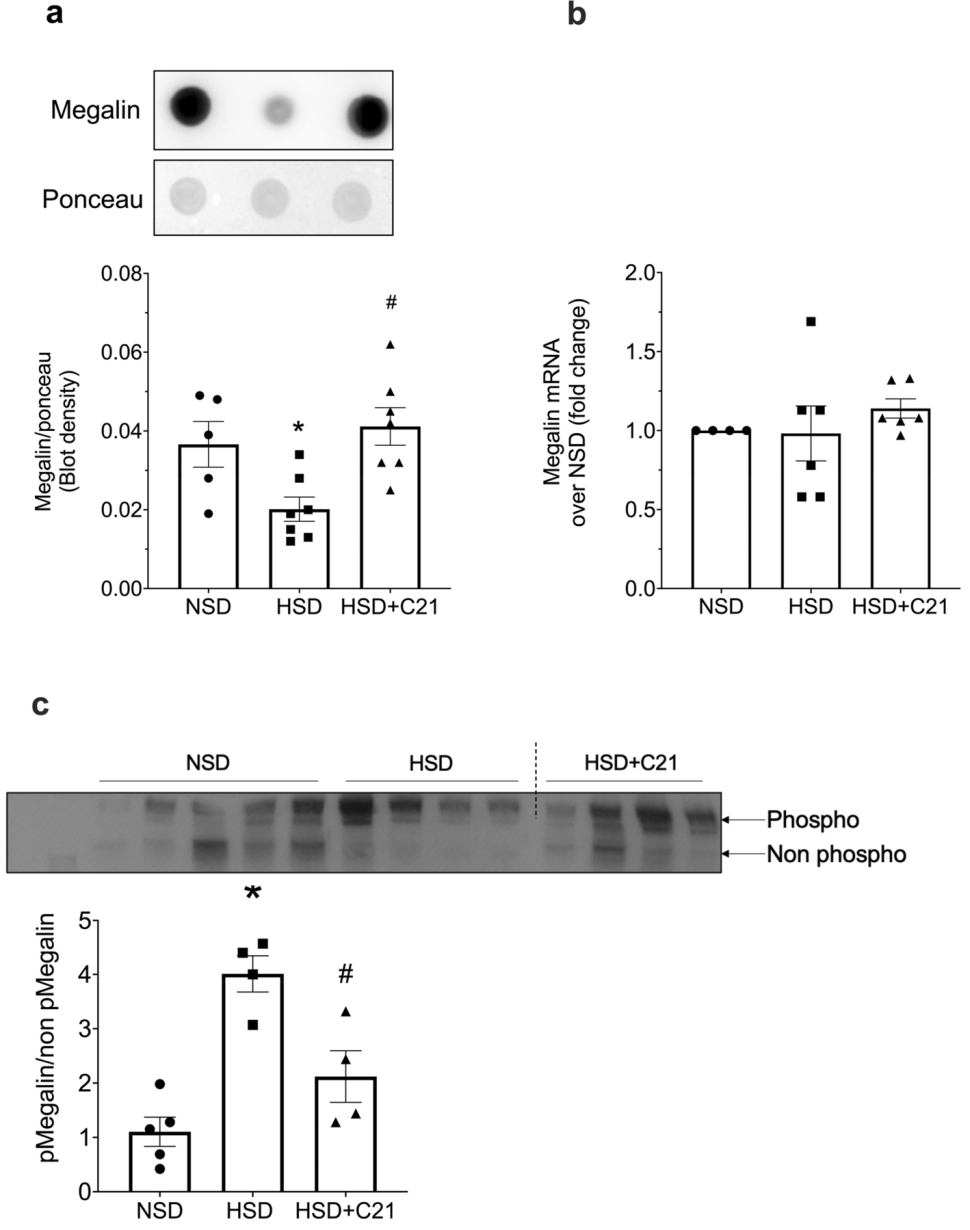
Expression of megalin through dot blot (**a**), mRNA expression of megalin (**b**) and western blot of phosphorylated and unphosphorylated-megalin (**c**). Densitometry was normalized by ponceau (**a**) and the ratio of phospho to non-phospho megalin expression on phostag gel is shown in 2c. Lanes 10 and 11 from Fig. 2c has been removed. Values are represented as mean ± sem; and one-way ANOVA followed by Fisher’s LSD test respectively (**b**, **c**). *p < 0.05 versus NSD and #*p* < 0.05 versus HSD.

**Figure 3:**
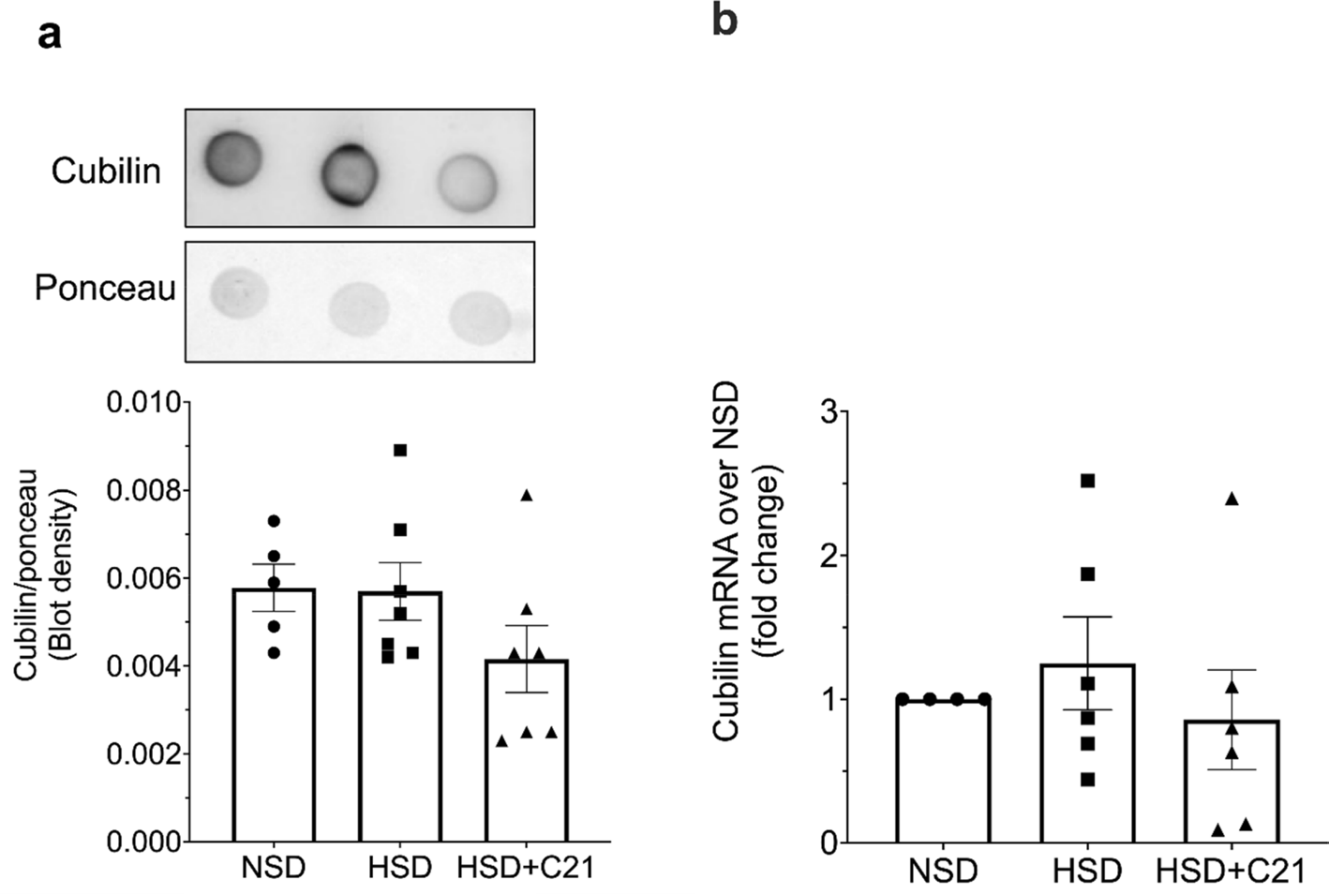
Expression of cubilin through dot blot (**a**) and mRNA expression of cubilin (**b**). Densitometry was normalized by ponceau (**a**). Values are represented as mean  +  sem; one-way ANOVA followed by Fisher’s LSD test respectively.

Densitometric analysis of dot blots revealed that HSD feeding caused a decrease in megalin expression and this decrease was prevented by C21 treatment (Fig. 2a). Megalin mRNA expression as measured by qPCR did not change in either HSD or HSD + C21 rats as compared to NSD rats (Fig. 2b). Densitometric analysis of phostag gel (western blot) revealed that HSD group exhibited significant increase in megalin phosphorylation as compared to NSD treatment group. Moreover, megalin phosphorylation was significantly reduced by C21 treatment compared with HSD alone (Fig. 2c). However, protein expression as measured by dot blot or mRNA of cubilin remained similar in all the three groups (Fig. 3a, b, respectively).

### Surface expression of megalin and cubilin through immunofluorescence

**Figure 4:**
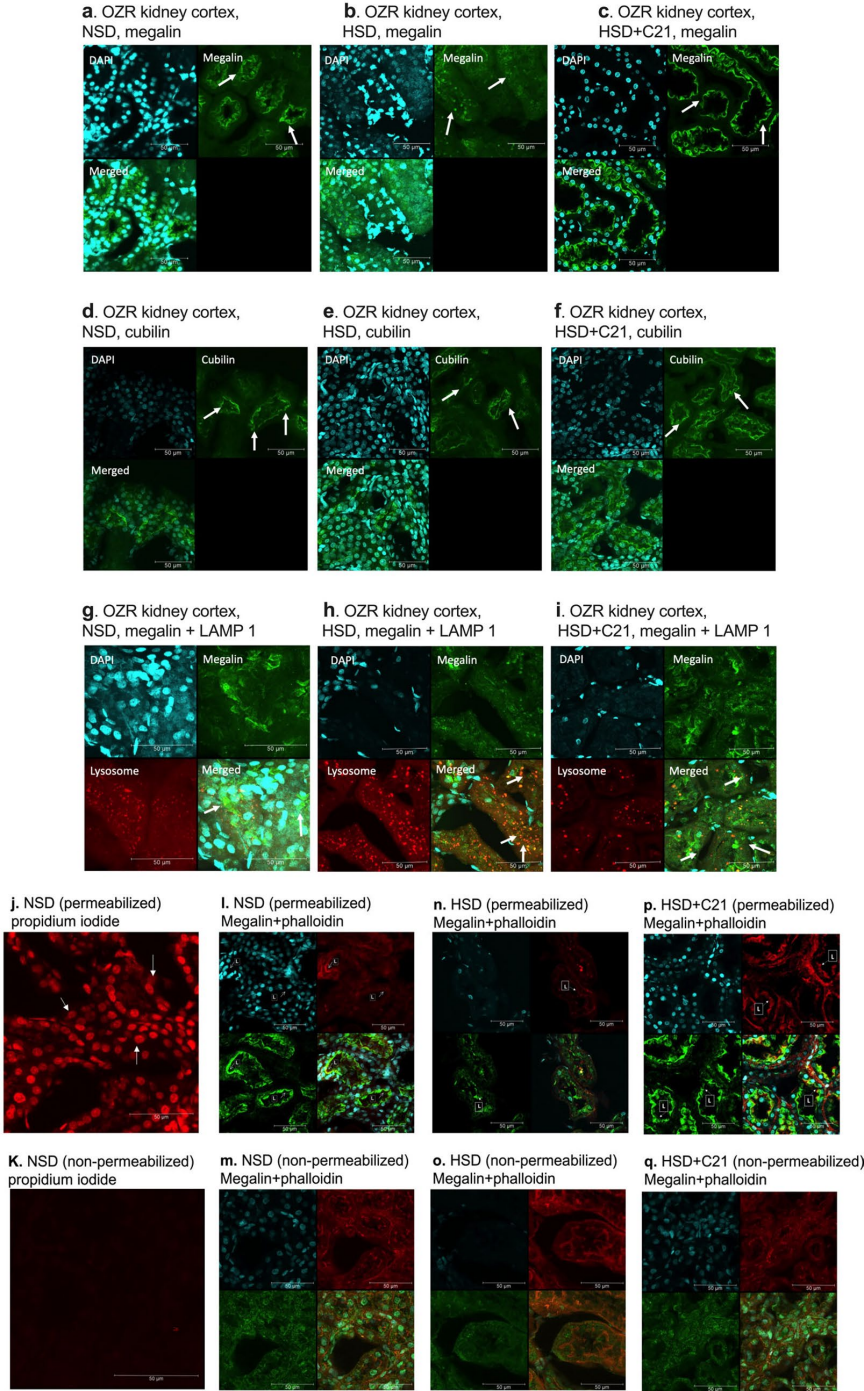
Representative images of cellular localization of megalin (**a**), cubilin (**b**) and megalin + LAMP1 (lysosomal marker) colocalization as determined by immunofluorescence in the kidney cortex. NSD- OZR fed with normal salt (0.4%, **a**, **d**, **g**), HSD-OZR fed with high salt (4%, **b**, **e**, **h**), and HSD + C21- OZR fed with high salt and treated with C21 (**c**, **f**, **i**). White arrows in (**a**, **b**, **c**) indicate megalin; (**d**, **e**, **f**) indicate cubilin and (**g**, **h**, **i**) indicate megalin + LAMP1. Kidney tissues (**j**, **l**, **n**, **p**) were permeabilized with saponin and tween 20 and (**k**, **m**, **o**, **q**) were not permeabilized. Kidney tissues (**j** and **k**) were stained with propidium iodide to demonstrate appropriate permeabilization. White arrows in panel “j” indicate nucleus. Images (**l**, **n**, **p**) indicate lumen (designated as “L” with white arrows) or apical membrane (red; we used phalloidin as the tubular plasma membrane marker) and megalin (green). All the images are of single plane as shown in figure S7. Scale 50 µm.

Confocal immunofluorescence microcopy revealed that in the NSD treated group, megalin was mostly present on the tubular cell surface (Fig. 4a). However, in the HSD-fed animals, megalin was found to be present mostly in the cytosolic compartment with negligible expression on the cell surface (Fig. 4b). Whereas treatment with C21 restored megalin localization back to the cell surface (Fig. 4c). Cubilin was found to be present on the tubular cell surface as well as in the cytosol of HSD treated rats whereas, in HSD + C21 treated and NSD fed rats, cubilin mostly was present on the cell surface of the tubules (Fig. 4d–f). Tissue permeabilization was validated using propidium iodide which is an impermeable nuclear dye in permeabilized kidney tissue (Fig. 4j) versus non-permeabilized kidney tissue (Fig. 4k). The tubular surface location was validated by phalloidin in permeabilized kidney tissue (Fig. 4l, n, p) versus non-permeabilized kidney tissue (Fig. 4m, o, q).

### Colocalization of megalin and lysosomal marker LAMP-1

LAMP-1 is a protein marker for lysosomes. Co-labeling of LAMP-1 and megalin revealed that megalin was co-localized with LAMP-1 in tubular cells of HSD-fed rats (Fig. 4h) and not in the NSD fed (Fig. 4g) or in HSD + C21 rats (Fig. 4i).

### Expression of glomerular injury markers

**Figure 5:**
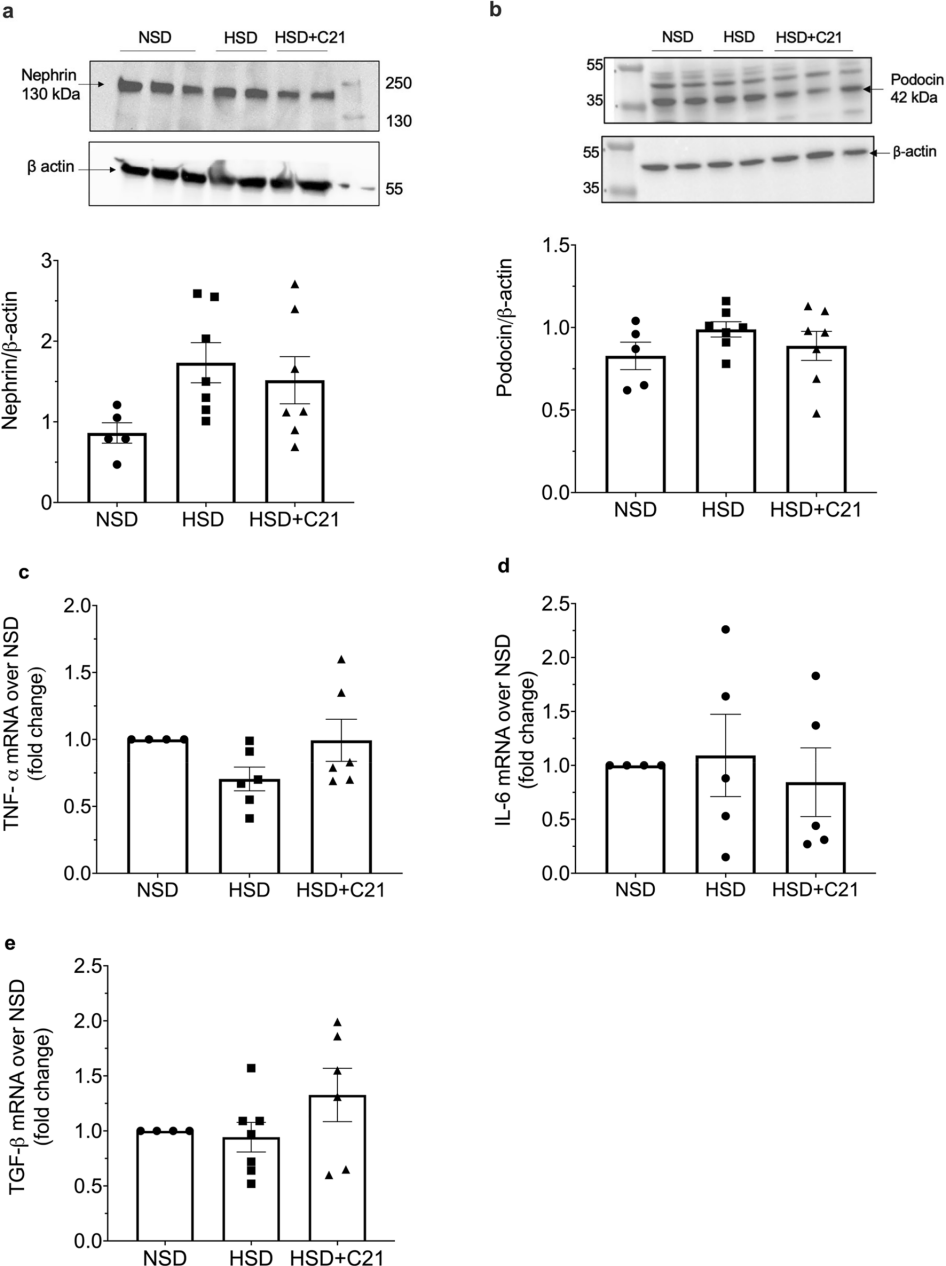
Representative western blots of expression of glomerular injury markers nephrin (**a**) and podocin (**b**) and the blots are used from the respective gels. Densitometry of the bands was normalized with β-actin expression. Quantitative mRNA analysis of TNF-α, IL-6 and TGF-β (**c**–**e**). Values are represented as mean ± sem; one-way ANOVA followed by Fisher’s LSD test.

The expression of nephrin and podocin (Fig. 5a and b respectively) as measured by western blot revealed no significant difference in HSD or HSD + C21 groups as compared to NSD group.

### Inflammatory and fibrotic markers

The inflammatory cytokines TNF-α (Fig. 5c), IL-6 (Fig. 5d) and the fibrotic marker TGF- β (Fig. 5e) were quantitated by measuring their mRNA and there was no difference in their levels between the NSD, HSD and HSD + C21 treatment groups.

### Akt/GSK3β activity

**Figure 6:**
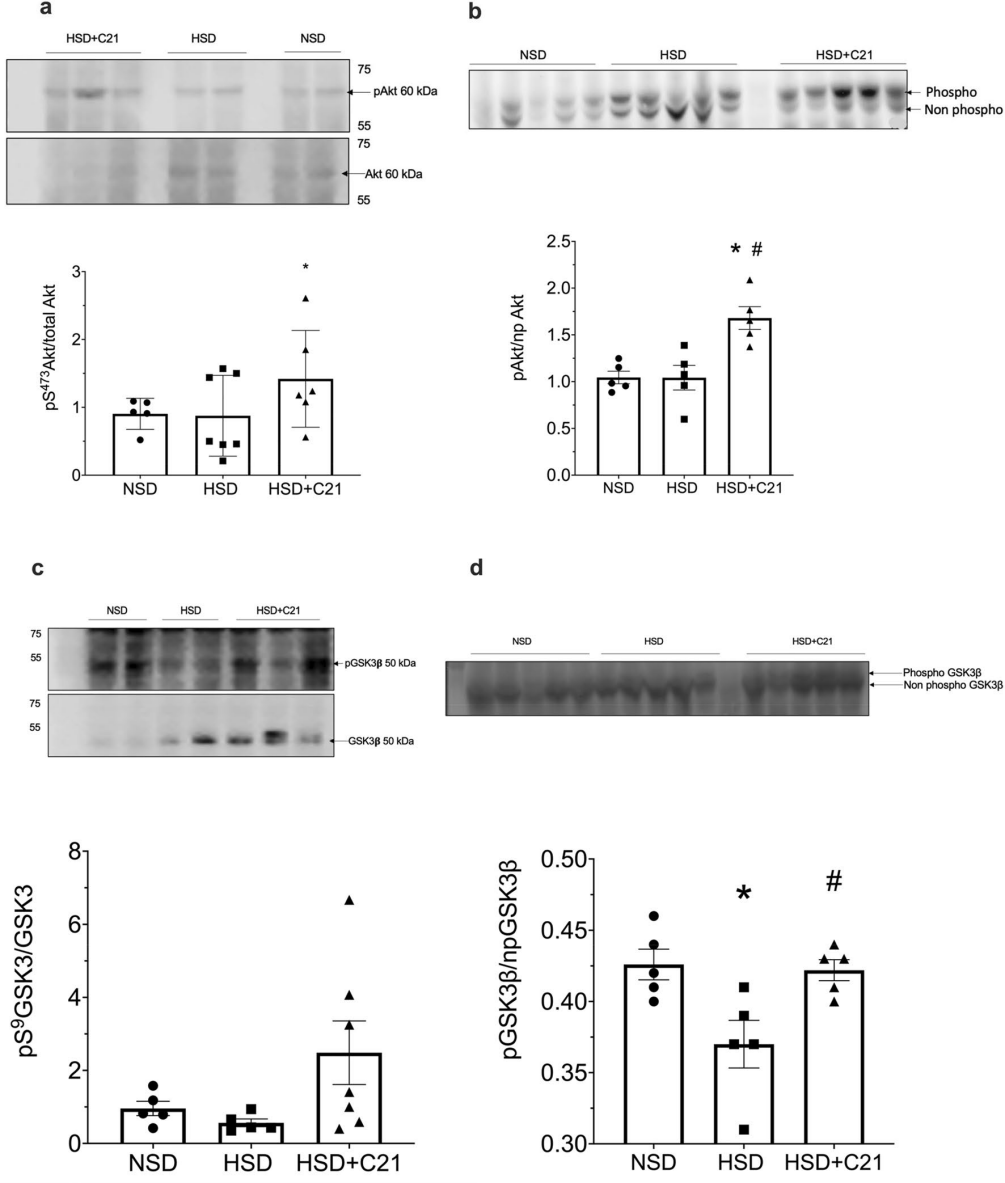
Representative western blot and phos-tag gel images of pAkt (6**a** and 6**b**) respectively and pGSK3 β (6**c** and 6**d**) respectively. Densitometry of phospho-serine bands was normalized total Akt and total GSK3 β (6**a** and 6**c**) respectively, the ratio of phospho- to non-phospho-Akt and GSK3 β respectively (6**b** and 6**d**). Values are represented as mean ± sem; one-way ANOVA followed by Fisher’s LSD test. *p < 0.05 versus NSD and #*p* < 0.05 versus HSD.

Akt phosphorylation as measured by p-S^473^-Akt antibody and Phostag western blotting with Akt antibody, was not affected in rats treated with HSD as compared to NSD group. Whereas Akt phosphorylation was significantly increased in HSD + C21 treatment group when compared to NSD and HSD groups (Fig. 6a and b). Similarly, GSK3β phosphorylation was measured by p-S^9^ GSK3β antibody and Phostag western blotting with GSK3β antibody. Both the methods revealed that HSD caused a modest decrease in the GSK3β phosphorylation, which was reversed in C21 + HSD group (Fig. 6c and d). However, the changes in GSK3β phosphorylation observed by p-S^9^ GSK3β antibody did not achieve statistical significance, although there was 44% decrease by HSD compared with NSD and 166% increase by C21 treatment.

## Discussion

**Figure 7:**
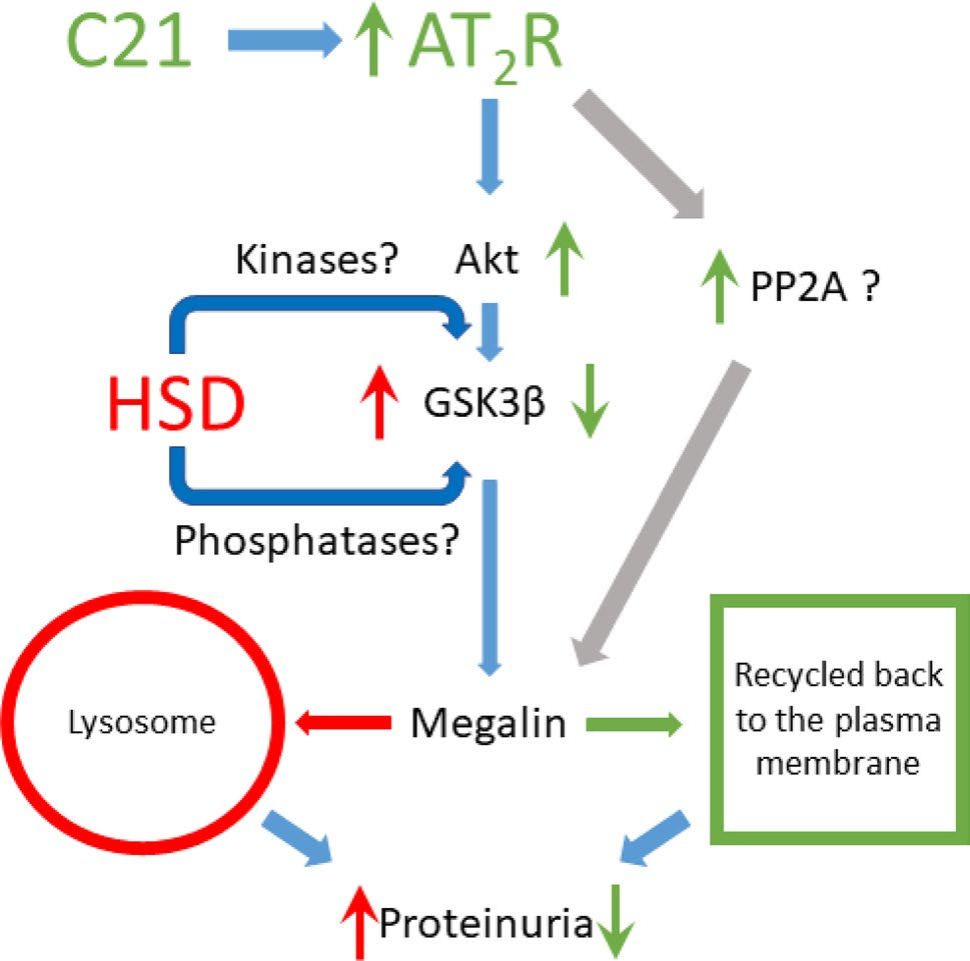
The schematic model supporting hypothesis.

This study investigates the early cellular and molecular mechanism of HSD-induced proteinuria and its protection by AT_2_R activation. Our data reveals that HSD intake causes proteinuria, which is associated with increased megalin phosphorylation and reduced cell surface expression in the kidney. Sub-cellular localization reveals the presence of megalin in the lysosomes suggesting that the reduced expression of megalin could be due to its degradation in the lysosomes. Also, non-phosphorylated (active) form of GSK3β which is a megalin phosphorylating enzyme is modestly decreased in HSD group. AT_2_R activation led to an increase in phosphorylated inactive form of GSK3β, reduction in megalin phosphorylation, prevention of lysosomal megalin localization, restoration of the surface megalin expression, and prevention of the onset of proteinuria. The AT_2_R activation enhances the Akt activity, which potentially might be responsible for reduction in GSK3β activity via its phosphorylation. The schematic model depicting the hypothesis and proposed mechanisms are provided in the Fig. 7.

Obesity is generally believed to be salt-sensitive in terms of kidney dysfunction, cardiovascular diseases, and hypertension^31,32^. High salt intake is known to be pro-fibrotic, pro-inflammatory and pro-oxidative stress^12,33,34^. Contrary to the high salt intake, the AT_2_R activation is anti-fibrotic and anti-inflammatory^2,5,35^. So, it can be argued that HSD may have caused damage to the tubules affecting megalin function through these processes, which are counteracted by AT_2_R activation thus restoring megalin recycling and function and improving proteinuria. However, our data reveals that 2 days of HSD feeding does not affect inflammatory markers (TNF-α and IL-6) and fibrotic marker (TGF-β) in the kidney. Nephrin and podocin are glomerular slit diaphragm proteins and their loss indicates glomerular injury. In this study, nephrin and podocin expression is not altered, so this rules out the possibility of a potential glomerular damage by HSD at 48 h. This data is further supported by the observation that GFR remained the same. Overall, this data suggests that renal damage or injurious processes i.e., inflammation, and fibrosis are unlikely to be the mechanisms responsible for proteinuria and disruption in megalin recycling to the cell surface in this early period of HSD feeding and that the protective effect of the AT_2_R agonist treatment in restoring megalin recycling and preventing proteinuria is likely the result of direct molecular and cellular effects. However, inflammation, fibrosis and structural and functional injury will increase by HSD intake if continued for a longer period as reported in obese rats^1^ and in normal mice placed on HSD^12^ Specifically, in obese rats, HSD feeding over 2-weeks period caused decrease in GFR, glomerular and tubular injury, infiltration of immune cells and fibrosis^12^. Also in normal mice, HSD feeding over 7-days period led to glomerular injury associated with inflammation and fibrosis^12^.

Megalin is clustered in clathrin coated pits and is delivered to early endosomes to recycle back to the plasma membrane^36,37^. Alteration in this cycle causes megalin to fuse with lysosome for degradation^29^ thus, likely reduces megalin surface expression and protein transport leading to proteinuria. GSK3β is one of the kinases which at unphosphorylated state is active and has been suggested to phosphorylate megalin and impair its recycling and reducing its cell surface expression^29^. In the present study, HSD causes a modest increase in activity of GSK3β (reduced phosphorylation) which may be responsible for increased megalin phosphorylation. However, it’s not known as to what have caused a reduction in GSK3β phosphorylation upon HSD intake. It is likely that an increase in megalin phosphorylation via GSK3β impaired megalin recycling and its lysosomal degradation, as we observed in our study that megalin is localized with the lysosomal marker  LAMP1. Since megalin mRNA remains unchanged in HSD group, this further supports the notion that it is the degradation, not the reduced synthesis, that leads to the overall decrease in megalin expression. The remaining megalin seems to be present mainly in the cytosol, not on the cell surface, which is necessary for its protein uptake/transport function. AT_2_R is known to activate Akt pathway^38^ and that Akt has a diverse function including phosphorylation of GSK3β leading to its inactivity^30,39^. In our study AT_2_R activation causes an increase in Akt phosphorylation. It is likely that AT_2_R activation reduced megalin phosphorylation via Akt/GSK3β pathway. This reduced phosphorylation may have prevented megalin trafficking toward lysosomes, and restored recycling process and megalin localization on the plasma membrane for its endocytic function. However, additional mechanisms, which are yet to be explored, may be involved in the impairment and restoration of megalin function in response to HSD and AT_2_R activation, respectively.

The reduced surface megalin expression can be a major mechanism of early increase in LMW proteinuria, while albuminuria, which makes < 10% of the total proteinuria in our study, remained unchanged. Since albumin and other high molecular proteins (> 68 kDa) makes a fraction of the filtered proteins in the absence of glomerular damage, it is likely that large proteins are effectively reabsorbed by cubilin with the remaining megalin. However, escaping of proteins from proximal tubule reabsorption and the subsequent passage through nephron can cause inflammation and fibrosis in the long-term causing tubulointerstitial and cardiovascular diseases. Our study reports that AT_2_R prevents megalin disruption presenting this as an early mechanism of HSD-induced proteinuria in HSD-fed obese rats and provides a basis for long-term beneficial effects as reported in several pre-clinical models of kidney diseases^1,40,41^.

### Summary

Overall this study provided molecular mechanisms associated with high salt-induced proteinuria and its reversal by AT_2_R activation, particularly independent of hypertension, inflammation and fibrosis which themselves are risk factors of proteinuria and kidney injury. Specifically, this study suggest that increased activity of GSK3β in response to HSD as a mechanism responsible for megalin recycling disruption and reduced expression. The reversal of this molecular process by AT_2_R activation via Akt pathway presents a potential mechanims of reducing HSD-induced protenuria with potentially protecting kidney injury in the long-term as has been reported earlier.

## Supplementary Information

Supplementary Figures.

## Data Availability

The data that support the findings of this study are available from the corresponding author upon reasonable request.
