# Supplementary material for: Angiotensin II type 2 receptor activation preserves megalin in the kidney and prevents proteinuria in high salt diet fed rats
Source: Sci Rep. 2023 Mar 15;13:4277. doi: 10.1038/s41598-023-31454-6 (PMC10017765; doi:10.1038/s41598-023-31454-6)
Supplement: Supplementary file 1 — Supplementary Figures. [file 41598_2023_31454_MOESM1_ESM.docx]

**Supplemental materials**

**Angiotensin-II type 2 receptor activation preserves megalin in the kidney and prevents proteinuria in high salt diet fed rats**

Kalyani Kulkarni, Sanket Patel, Riyasat Ali, Tahir Hussain*

Department of Pharmacological and Pharmaceutical Sciences, College of Pharmacy, University of Houston, Houston TX 77204

Running headline: AT_2_R activation preserves renal megalin

**Correspondence:**

Tahir Hussain

Address: University of Houston, College of Pharmacy, Health 2, 4349 MLK Blvd., Health 2, Houston, TX 77204-5037

Office: 713-743-1276

Fax: 713-743-1884

Email: thussain@central.uh.edu


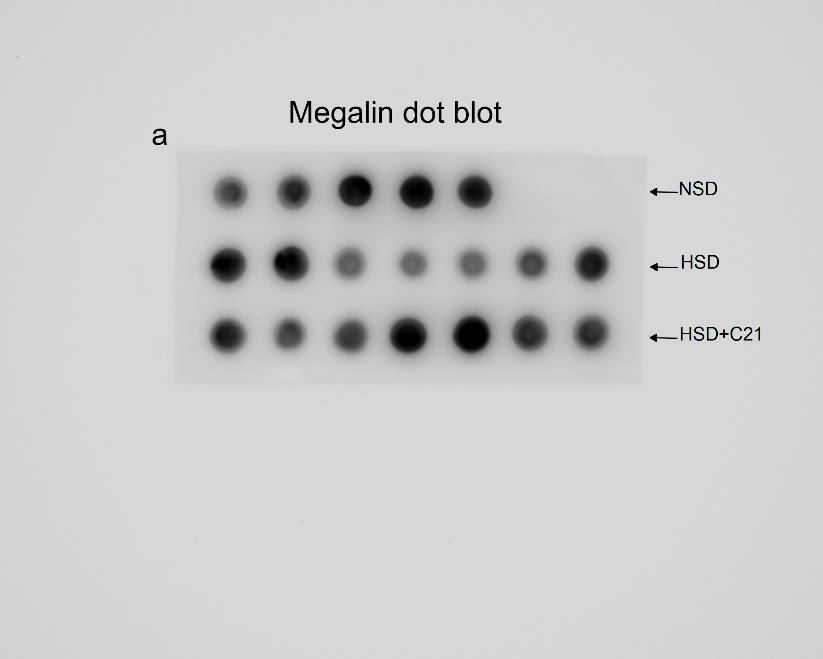

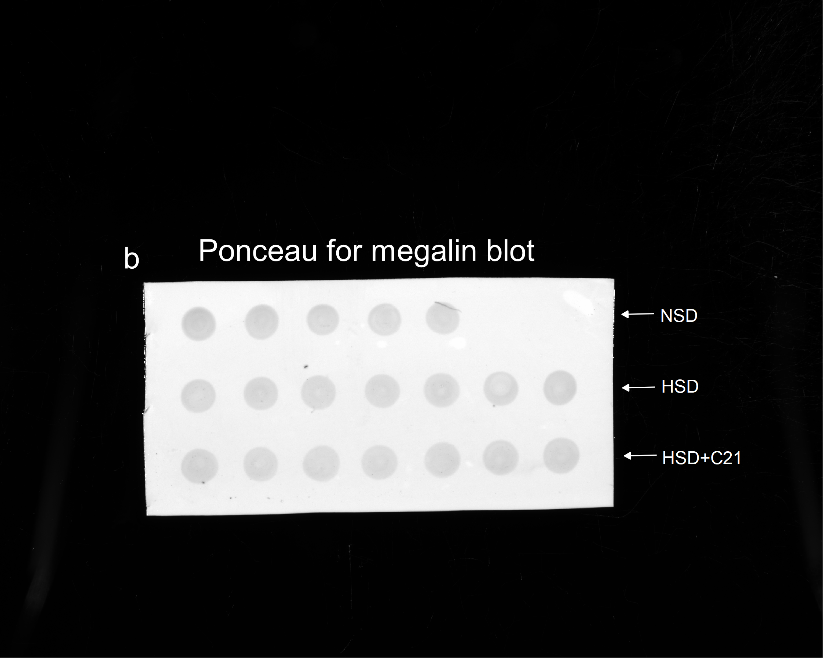


**Figure S1:** Dot blot membranes of megalin (a) and ponceau (b). NSD was loaded into column 1, HSD was loaded into column 2 and HSD+C21 was loaded into column 3.


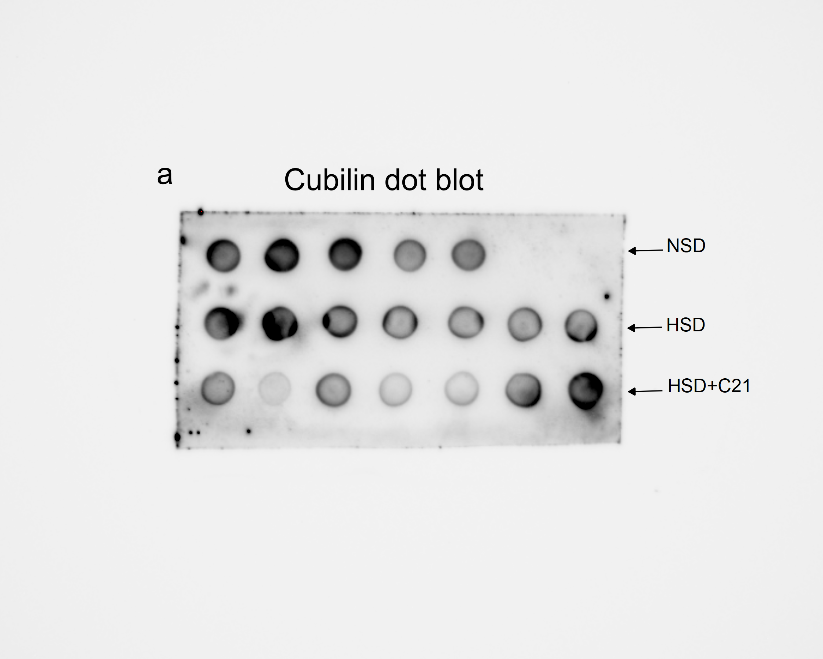

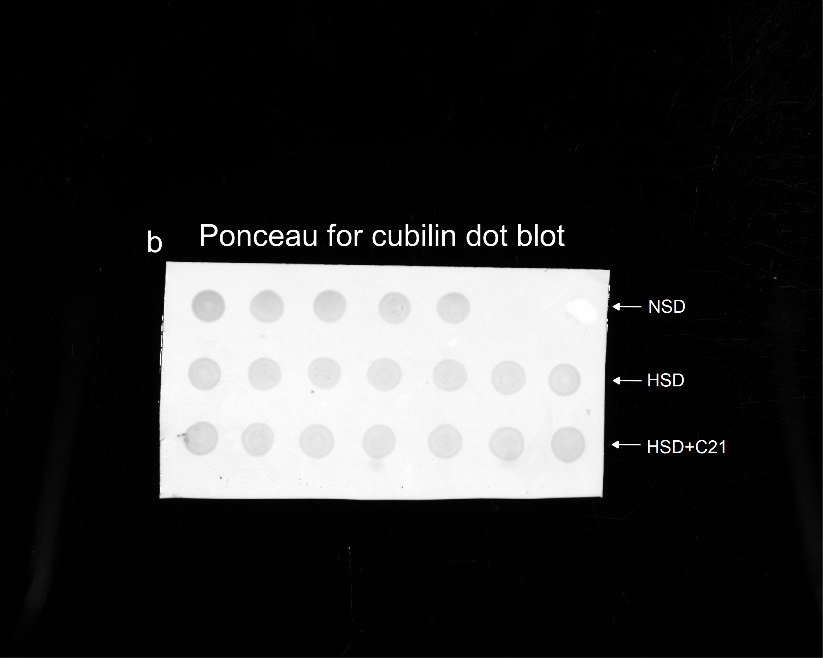


**Figure S2:** Dot blot of membranes of cubilin (a) and ponceau (b). NSD was loaded into column 1, HSD was loaded into column 2 and NSD was loaded into column 3.


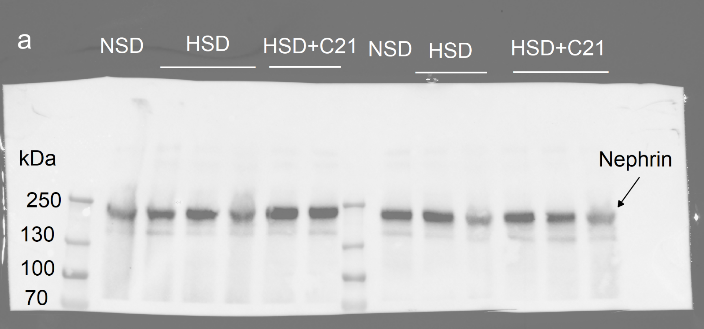

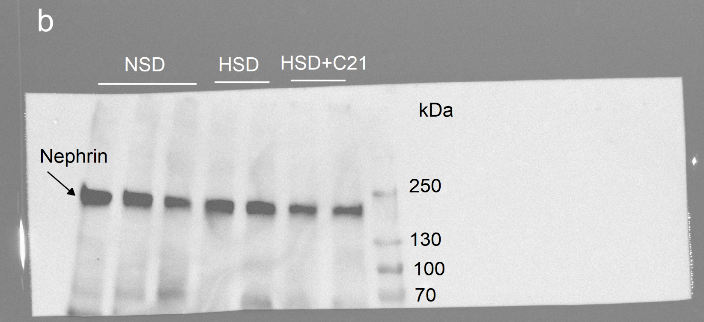


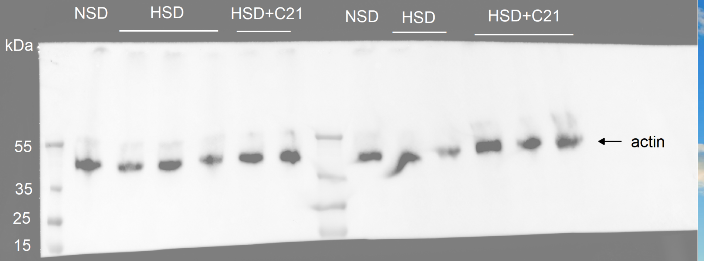

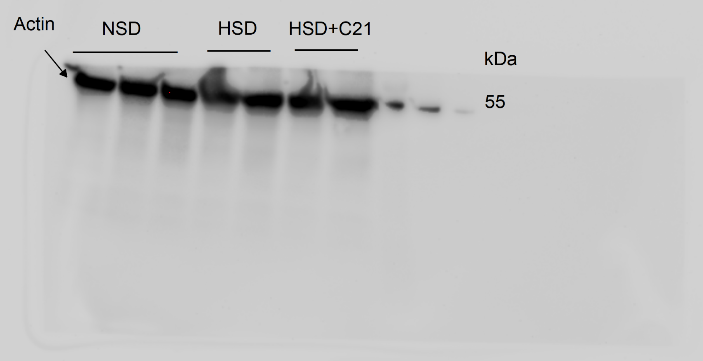


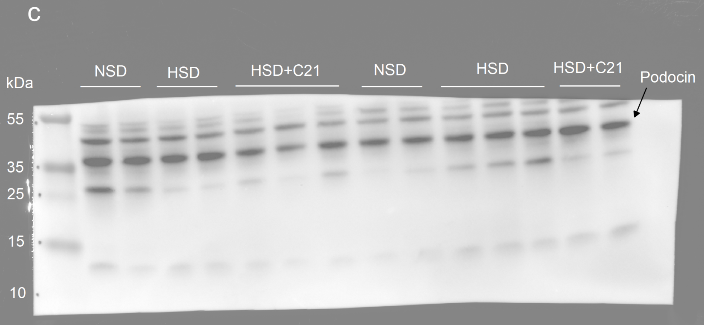

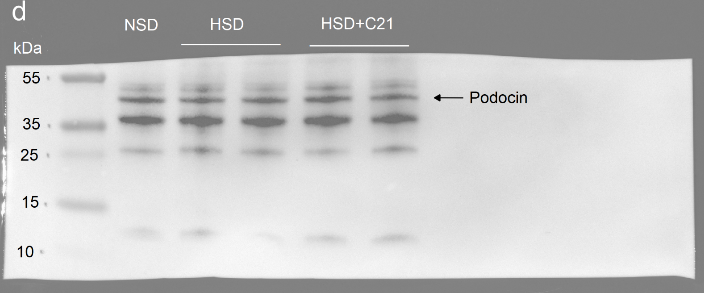


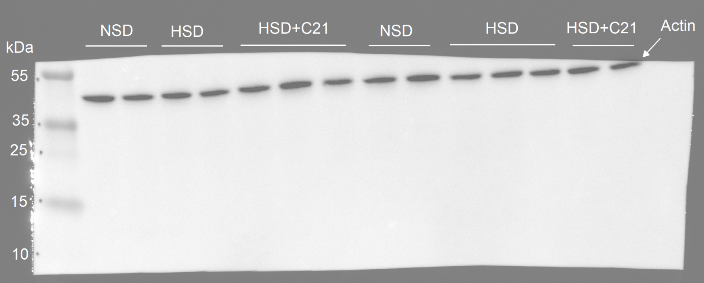

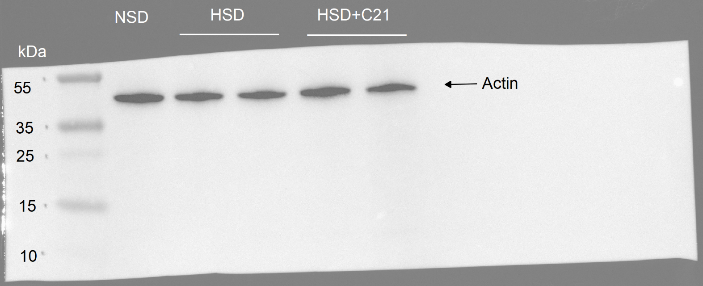


**Figure S3:** Western blot membranes of nephrin and podocin. Protein ladder was loaded into lanes 1and 8 of membranes a, c and d and into lane 7 of membrane b. NSD was loaded into lane 2 and 8 of membrane A and into lanes 1, 2, 3 of membrane b. NSD was loaded into lanes 2, 3 and 9, 10 of membrane c and into lane 2 of membrane d. HSD was loaded into lanes 3, 4, 5, 11, 12 of membrane a, into lanes 4, 5 of membrane b, into lanes 4, 5 and 11, 12, 13 of membrane c and into lanes 3,4 of membrane d. HSD+C21 was loaded into lanes 6, 7 and 13, 14, 15 of membrane a, into lanes 6, 7 of membrane b, into lanes 6, 7, 8 and 14, 15 of membrane c and into lanes 5, 6 of membrane d.


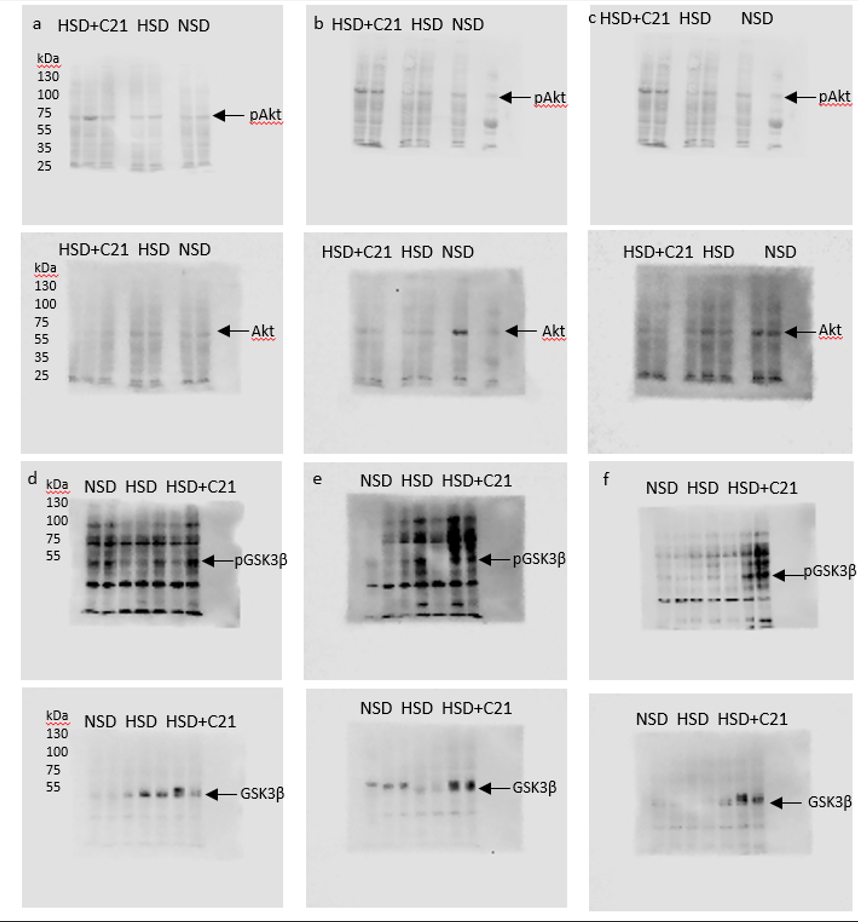


**Figure S4:** Western blot membranes of pAkt, total Akt, pGSK3β and total GSK3β. Protein ladder was loaded into lanes 4, 7, 10 of membrane a, lanes 3, 6, 10 of membrane b, lanes 3, 6, 10 of membrane c, and lane 1 of membranes d, e, f. NSD was loaded into lanes 8, 9 of membrane a, lane 8 of membrane b, 8, 9 of membrane c, and into lanes 2, 3 of membranes d, e, f. HSD was loaded into lanes 5, 6 of membrane a, 4, 5 of membrane b, 4, 5, 6 of membrane c, 4, 5 of membrane d, 4, 5 of membrane e and into lanes 4, 5, 6 of membrane f. HSD+C21 was loaded into lane 1, 2, 3 of membrane a, 1, 2 of membrane b, 1, 2 of membrane c, 6, 7, 8 of membrane d, 6, 7 of membrane e and 7, 8 of membrane f.


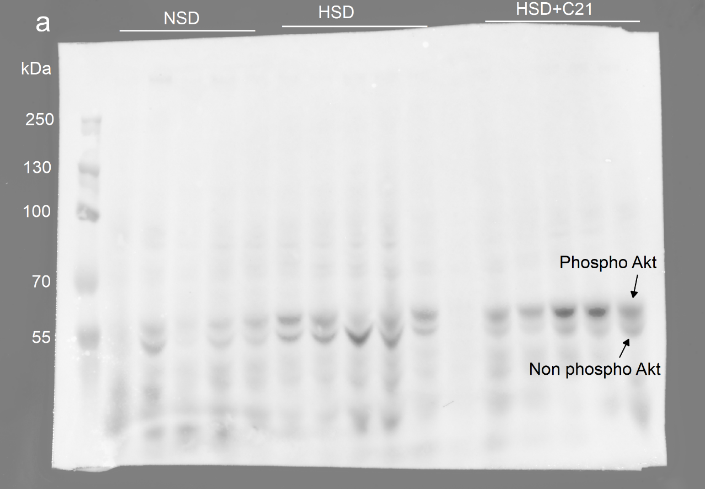

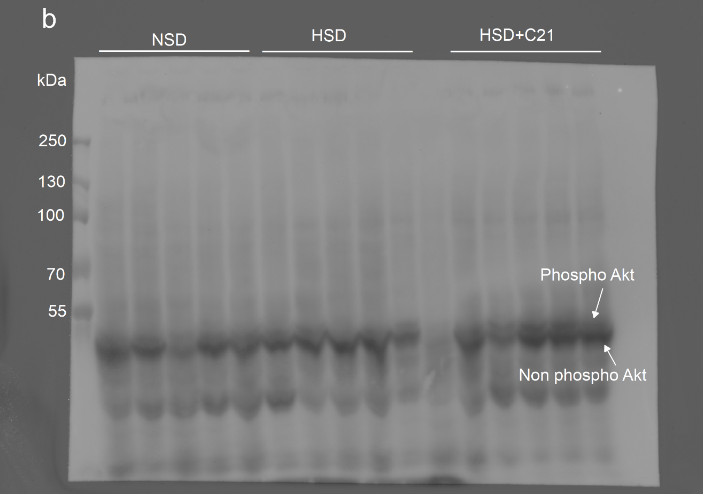

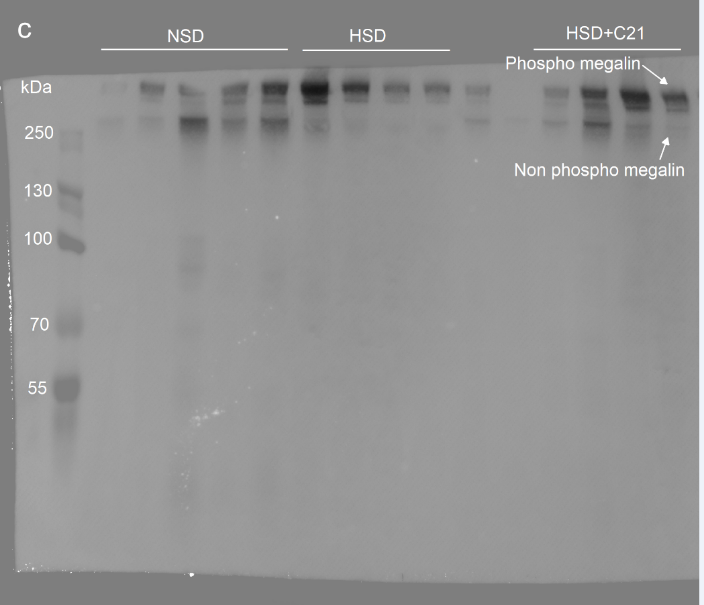


**Figure S5:** Phostag membranes of Akt, GSK3β and megalin (a, b, c) respectively. Protein ladder was loaded into lane 1 of membranes a, b, c. NSD was loaded into lanes 2-6 of all three membranes. HSD was loaded into lanes 7-11 of membranes a and b and into lanes 7-10 of membrane c. HSD+C21 was loaded into 13-17 lanes of a and b membranes and into 13-16 lanes in membrane c.


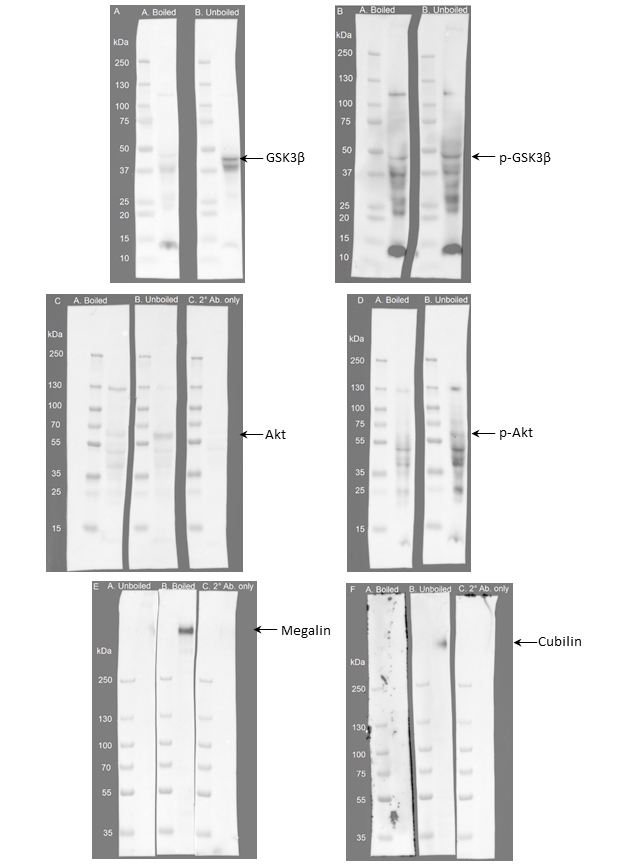


**Figure S6.** Validation of primary antibody of phospho-GSK3β (A), GSK3β (B), phospho-Akt (C), Akt (D), megalin (E), and cubilin (F) using male obese Zucker rat kidney homogenate and incubating it traditionally with boiled (95°C, 5 minute) antibody (left, A-F) and unboiled (middle, A-F) primary antibody (overnight, 4°C, gentle rotation) followed by incubation with the respective secondary (2°) (anti-rabbit, A-D; anti-mouse, E; anti-goat, F) antibody (ab.) (1 hour, room temperature, gentle rotation). Some PVDF membranes were incubated with 2° antibody only (right; 2° anti-rabbit = C; 2° anti-mouse = E; 2° anti-goat ab. = F). Membranes were washed in-between incubation steps with PBS (3X10’). The membranes were placed together and chemiluminescence signal was acquired via ChemiDoc MP system (BioRad).

**For all western blots within the manuscript and SI figures:**

Images are not processed or manipulated. Images were only adjusted for brightness and contrast using only the BioRad ChemiDoc MP or Li-Cor Odyssey Fc software. Brightness and contrast is equally applied to all images. Aspect ratio has not been altered for any images.





Figure S7. The orthogonal (XZ and YZ) localization of megalin (a-c), cubilin (d-f), and colocalization of megalin and LAMP1 (g-i) in the kidney sections of obese Zucker rat (the actual Z-stacked images are provided in Figure 4 of original manuscript).

**For Figure 4 and S7:**

Images are provided at the resolution collected. No adjustment is applied. Threshold manipulation, expansion or contraction of signal or the altering of high signal is not done. Pseudo-coloring and nonlinear adjustments are not used. Adjustment of individual color channel is not done. Fluorochrome excitation and emission settings are inbuilt within the Leica TCS SP8 CLSM and are not manipulated. Images are not processed; deconvolution is not applied. The images are of single plane.
